# Clinical Utilization and Performance of Bempedoic Acid in an Italian Real-World Setting: Insight from Campania Region

**DOI:** 10.3390/jcm14061839

**Published:** 2025-03-09

**Authors:** Vincenzo Russo, Gennaro Ratti, Antonio Parrella, Aldo De Falco, Mario Crisci, Riccardo Franco, Giuseppe Covetti, Alfredo Caturano, Giovanni Napolitano, Fortunato Scotto di Uccio, Gennaro Izzo, Luigi Argenziano

**Affiliations:** 1 Cardiology Unit, Department of Medical Translational Sciences, University of Campania “Luigi Vanvitelli”—Monaldi Hospital, 80131 Naples, Italy; aldodefalco@yahoo.it; 2 Cardiology Unit, San Giovanni Bosco Hospital, Health Authority Naples 1, 80144 Naples, Italy; genratti@virgilio.it; 3 Department of Medicine and Medical Specialties, A. Cardarelli Hospital, 80131 Naples, Italy; antonio.parrella@aocardarelli.it (A.P.); giuseppe.covetti@aocardarelli.it (G.C.); 4 Cardiology Unit, Department of Cardiology, Monaldi Hospital, 80131 Naples, Italy; mario.crisci1984@gmail.com; 5 Cardiology Unit, San Giuliano Hospital, Health Authority Naples 2 North, 80014 Naples, Italy; dottorriccardofranco@gmail.com (R.F.); gv.napolitano@gmail.com (G.N.); 6 Department of Human Sciences and Promotion of the Quality of Life, San Raffaele Roma Open University, 00166 Rome, Italy; 7 Cardiology Unit, Ospedale del Mare ASL NA1 Centro, 80147 Naples, Italy; scottof@libero.it (F.S.d.U.); gennaroizzo1982@gmail.com (G.I.); 8 Cardiology Unit, Pineta Grande Hospital, 81030 Castel Volturno, Italy; luigi.argenziano@pinetagrande.it

**Keywords:** bempedoic acid, lipid-lowering therapy, low-density lipoprotein cholesterol (LDL-c), persistence, adherence, side effects, cardiovascular risk

## Abstract

**Background/Objectives**: Bempedoic acid (BA) is a novel lipid-lowering agent that reduces low-density lipoprotein cholesterol (LDL-c) and cardiovascular events. Limited real-world data on its effectiveness and safety are available. This study aimed to evaluate the utilization and clinical performance of BA in routine clinical practice. Moreover, an explorative pharmacoeconomic analysis was performed. **Methods**: We prospectively enrolled consecutive patients with dyslipidemia who started 180 mg BA, alone or with 10 mg ezetimibe, across five outpatient clinics in Campania Region, Italy from September to December 2023. Clinical and laboratory assessments, including lipid profile, hepatic function, and creatine phosphokinase levels, were performed at baseline and at least after one month follow-up. Side effects were recorded. **Results**: 111 patients (age 65 ± 9 years, 61% male) were included. At BA initiation, 70.3% were on maximally tolerated statin dosage and ezetimibe, 16.2% on ezetimibe alone, and 13.5% on PCSK9 inhibitors due to statin intolerance. BA significantly reduced LDL-c serum levels (89.9 ± 33.0 vs. 56 ± 27.6 mg/dL; *p* < 0.0001), with 46% achieving therapeutic targets. LDL-c decreased by 28% in patients on intensive statins/ezetimibe and by 45% in statin-intolerant patients, with reduced healthcare costs. Side effects were infrequent (10%) and reversible. Adherence was 99%, and persistence 90%. **Conclusions**: In our clinical pratice, BA was primarily used in high-risk patients with dyslipidemia who failed to reach LDL-c therapeutic target with statins/ezetimibe, and to a lesser extent, in statin-intolerant individuals. BA treatment enabled 54% to reach LDL-c therapeutic target. BA was well tolerated, and showed high adherence and persistence, contributing to cost savings.

## 1. Introduction

Bempedoic acid (BA) is a novel lipid-lowering agent that exerts its effect by inhibiting the enzyme adenosine triphosphate-citrate lyase (ATC or ACLY), blocking the mevalonate pathway of cholesterol synthesis, upstream of 3-hydroxy-3-methylglutaryl-coenzyme A reductase, which is the target of statins [1]. Its complementary mechanism of action, distinct from statins, allows for additional low-density lipoprotein cholesterol (LDL-c) reduction when combined with other lipid-lowering therapies [1]. Several clinical evidences suggest that a substantial proportion of patients, ranging from 20% to 30%, in various cohorts fail to reach guideline-recommended LDL-c therapeutic targets with statins alone, reinforcing the need for alternative treatments such as BA [2,3]. The improved LDL-c control provided by BA leads to a lower risk of major cardiovascular outcome events; moreover, BA demonstrates a generally favorable safety profile, with the exception of an elevated risk of gout [4,5,6,7]. BA was recently approved in Italy for the treatment of hypercholesterolemia in subjects with statin intolerance or those who are unable to reach the LDL-c therapeutic target despite receiving the maximal tolerated lipid-lowering therapy [8,9]. Few data are available about the clinical performance of BA in real-world settings [10,11]. The aim of this study was to assess the clinical utilization of BA in our clinical practice and to evaluate its clinical performance among real-world patients; moreover, an explorative pharmacoeconomic analysis was performed.

## 2. Materials and Methods

### 2.1. Study Population

We included all consecutive patients diagnosed with dyslipidemia, unresponsive to dietary modifications and physical activity, who started 180 mg BA, either as monotherapy or in fixed combination with 10 mg ezetimibe, followed at five outpatient clinics in Campania Region, Italy, from September 2023 to December 2023. Patients with documented dyslipidemia who met clinical criteria for BA therapy (statin intolerance or inadequate LDL-c reduction despite maximal tolerated lipid-lowering therapy) were eligible to be included in the present study. Exclusion criteria were age lower than 18 years, pregnancy or breastfeeding, known hypersensitivity or contraindications to BA, significant hepatic dysfunction (ALT or AST serum levels > 3 × the upper limit of normal), severe renal impairment (eGFR < 30 mL/min/1.73 m^2^).

### 2.2. Study Protocol

This is a multicenter, prospective observational cohort study designed to reflect routine clinical practice. At baseline, all participants underwent a comprehensive evaluation including medical history, physical examination, 12 lead electrocardiogram (ECG) and laboratory assessment; which were repeated at follow-up after an uninterrupted 180 mg BA therapy, alone or in combination with 10 mg ezetimibe (BA + Eze). 

The laboratory assessment included total cholesterol (TC), LDL-c, high-density lipoprotein cholesterol (HDL-c), triglycerides (TG), alanine aminotransferase (ALT), aspartate aminotransferase (AST), creatinine, urate, and creatine phosphokinase (CPK) serum levels. Blood tests, collected after an 8-h overnight fast, were performed at baseline, before treatment initiation, and at least one month after the administration of the study drug. A pill count (number of doses taken) was performed. All side effects leading to temporary or definitive suspension were reported. All patients provided written informed consent for data storage and analysis. The study was conducted in accordance with the Declaration of Helsinki and its subsequent amendments, and it was approved by the local ethics committee (Ethical Committee of University of Campania “Luigi Vanvitelli”—Monaldi Hospital; ID 23263/2022; approval date: 15 December 2022).

### 2.3. Study Objectives

The objectives of this study were as follows: (1) to report the clinical utilization of BA in our routine clinical practice; (2) to assess the effectiveness and safety of BA treatment; (3) to evaluate adherence and persistence to BA therapy; (4) to perform an exploratory pharmacoeconomic evaluation of BA use in a real-world setting.

The effectiveness was evaluated based on the reduction in LDL-c serum levels at follow-up among overall population and across patient subgroups. Treatment adherence was measured using the pill count method, calculated as the number of doses taken divided by the number of doses dispensed from the last prescription, multiplied by 100 (%). Persistence to therapy was defined as the absence of any temporary or permanent interruption due to side effects.

### 2.4. Statistical Analysis

Categorical data were presented as frequency and percentage, while continuous variables were expressed as either median (interquartile range [IQR]) or mean ± SD, depending on their distribution, which was assessed using both the Kolmogorov–Smirnov and the Shapiro–Wilk tests. Differences for categorical variables were assessed by the chi-square test, with the application of Yates correction where appropriate. Either parametric Student’s *t*-test or nonparametric Mann–Whitney U test and Wilcoxon test were used to compare continuous variables, depending on their distribution. Box plot analysis was performed to visually depict LDL levels within each therapy group at baseline and after BA add-on. A 2-sided *p*-value < 0.05 was considered statistically significant. All analyses were conducted using SPSS software version 24.0 (SPSS, Chicago, IL, USA), while box plots were generated using RStudio^®^ 2024.09.1 software (RStudio, Boston, MA, USA).

## 3. Results

111 consecutive patients (age 65 ± 9 years, 61% male) with dyslipidemia not achieving LDL-c therapeutic target with available lipid-lowering therapies were enrolled. The baseline characteristics of the study population are showed in Table 1. According to the European Society of Cardiology Guidelines, 98 patients (88%) were at very high cardiovascular risk, while 13 patients (12%) were at high cardiovascular risk [12].

At the BA prescription, 78 patients (70.3%) were on maximum tolerated statins dosage and ezetimibe, of whom 53 patients (67.9%) were on 20 mg rosuvastatin (Rosu/Eze group) and 25 (32.1%) were on 40 mg atorvastatin (Ato/Eze group); 18 (16.2%) were on 10 mg ezetimibe alone; 15 patients (13.5%) were on PCSK9i therapy for at least six months. Finally, 67 patients (60.3%) started BA alone therapy, and 44 patients (39.7%) started the fixed combination of BA and ezetimibe.

### 3.1. Follow-Up

At a median follow-up of 60 days (IQR 44.5–60 days), 10 patients (9.0%) discontinued BA therapy due to musculoskeletal complaints (*n* = 4, 3.6%), gastrointestinal complaints (*n* = 3, 2.7%), or hyperuricemia (*n* = 3, 2.7%). The study population cohort exhibited a statistically significant reduction in LDL-c serum level (89.9 ± 33.0 vs. 56 ± 27.6 mg/dL; *p* < 0.0001), TC (155.5 ± 36.7 vs. 124.3- ± 31.1 mg/dL; *p* < 0.0001), and non-HDL cholesterol (97.0 ± 41.6 vs. 80.2- ± 28.0 mg/dL; *p* < 0.0001). While no significant variations were observed in TG (127.3 ± 57.4 vs. 119.3 ± 39.0 mg/dL; *p* = 0.7) and HDL-c (51.1 ± 16.9 vs. 44.1 ± 11.4 mg/dL; *p* = 0.1) plasma levels. A statistically significant increase with no clinical relevance was shown in AST (33.2 ± 10.5 vs. 39.2 ± 14.4 UI/dL; *p* = 0.002), ALT (36.7 ± 13.2 vs. 40.3 ± 16.3 UI/dL; *p* = 0.06), and uric acid (5.1 ± 1.4 vs. 5.4 ± 1.3 mg/dL; *p* = 0.014) serum levels. CPK serum levels (141.3 ± 147.7 vs. 136.8 ± 87.7 UI/dL; *p* = 0.7) remained stable over time. No temporary interruptions were reported throughout the study. The adherence rate to the therapy regimen reached 99%, demonstrating high treatment compliance.

### 3.2. Subgroup Analysis

**Figure 1:**
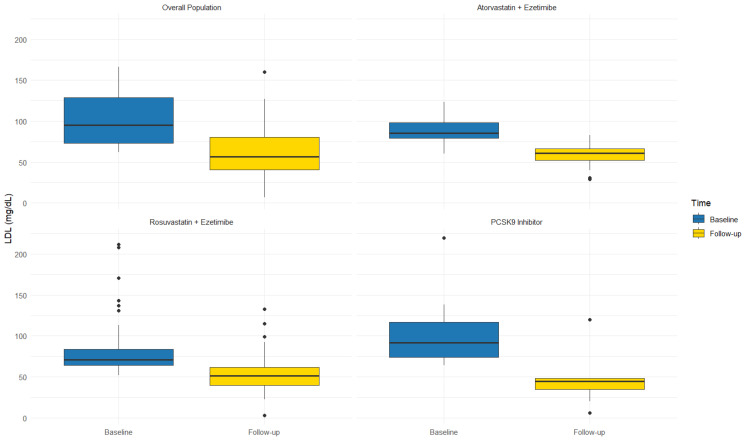
LDL levels across different lipid-lowering therapies at baseline and after bempedoic acid add-on.

In Rosu/Eze group (*n* = 53), adding BA therapy resulted in a 29.8% reduction in LDL-c (77.8 ± 31.0 vs. 54.6 ± 25.9 mg/dL; *p* < 0.0001) (Figure 1), with 60.4% patients (*n* = 32) achieving the appropriate LDL-c therapeutic target for their risk level. Similarly, in the Ato/Eze group (*n* = 25), adding BA therapy resulted in a 26.3% reduction in LDL-c (77.5 ± 33.2 vs. 57.1 ± 24.7 mg/dL; *p* < 0.019) (Figure 1), with 36% patients (*n* = 9) achieving the appropriate LDL-c therapeutic target for their risk level. Moreover, the add-on therapy with BA allowed 53% of patients on high-intensity statins and ezetimibe at both high and very high CV risk to achieve their LDL-c therapeutic target. Among patients on PCSK9i therapy (*n* = 15), the use of BA/Eze fixed combination therapy resulted in a 50% LDL-c reduction (93.0 ± 26.1 vs. 46.9 mg/dL ± 26.2 mg/dL; *p* < 0.0001) (Figure 1), with 60% of them (*n* = 9) reaching the LDL-c therapeutic target at follow-up. Finally, in patients on ezetimibe monotherapy (*n* = 18), adding BA resulted in a 42.0% reduction in LDL-c (83.9 ± 21.8 vs. 48.6 ± 20.4 mg/dL; *p* < 0.0001), with 56.5% of them (*n* = 10) reaching the LDL-c therapeutic target.

### 3.3. Pharmacoeconomic Analysis

We evaluated two treatment scenarios for 78 patients who had not achieved their LDL-c therapeutic target with background therapy and were treated with BA as an add-on therapy. In Scenario 1 (real world) we calculated the annual treatment cost by determining the number of patients reaching their target with 12 months of ezetimibe + statins + BA (*n* = 41). For the patients who did not achieve their target (*n* = 37), we considered the cost of one month of ezetimibe + statins + BA, followed by 12 months of treatment with ezetimibe + statins + PCSK9i. In Scenario 2 (pre-BA era), we calculated the cost assuming all 78 patients would have been treated with ezetimibe + statins + PCSK9i for the entire year. Prices considered include those published on the web platform of Campania Regional Society for the healthcare service (SO.RE.SA) [13], the Italian Medicine Agency (AIFA) transparency lists [14] for first and second-level treatments, and the prices published in the Official Gazette of the Italian Republic [15] for BA.

**Figure 2:**
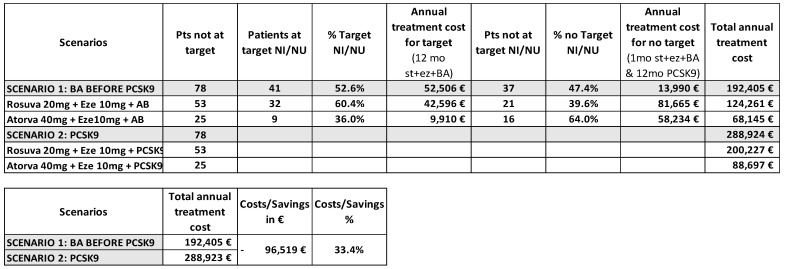
Pharmacoeconomic analysis of BA treatment in patients not achieving their LDL-c therapeutic target with background therapy. Scenario 1 describes the real-world setting; Scenario 2 describes the optimal approach in pre-BA era.

In Scenario 1 the use of BA as an add-on therapy before introducing PCSK9i resulted in a total annual treatment cost of €192,405. In Scenario 2, where all 78 patients would have been treated with PCSK9i for the entire year, the total annual cost was €288,924. The triple oral lipid lowering therapy, before introducing PCSK9i, therefore yielded savings of €96,519, representing a 33.4% reduction compared to Scenario 2 (Figure 2).

## 4. Discussion

The key findings of our study are the following: in our clinical practice, BA was mainly prescribed to patients with dyslipidemia who were not achieving LDL-c therapeutic target despite maximum tolerated statin dosage and ezetimibe treatment, and to one-third of the patients with statin intolerance. BA resulted in a 28% reduction in LDL-c serum levels for patients on intensive statins and ezetimibe, and a 45% reduction for statin-intolerant patients. Among the overall population, 54% of patients receiving BA achieved the LDL-c therapeutic target after at least one month of treatment, accompanied by a reduction in regional healthcare spending. BA was safe, well-tolerated, and demonstrated high adherence and persistence.

While few and contrasting data are available about the effectiveness and safety of BA in real-world settings, none of these studies have been conducted in European countries [10,11], and no data are yet available on the utilization of BA in Italian clinical practices.

In a retrospective study [10] including 73 American patients with increased risk for atherosclerotic events and statin intolerance, BA was linked to clinically significant LDL-c reduction (LDL-c −36.7%, −31%, and −20.3% at ≤3, 6, and 12 months, respectively). However, a significant incidence of emergent adverse events (32.8%), primarily musculoskeletal complaints, led to drug discontinuation, was reported. In a real-world open-label study [11] including 122 Indian patients with acute coronary syndrome, the combination of 40 mg rosuvastatin, ezetimibe, and BA, initiated at hospital admission, resulted in a 60.6% reduction in LDL-c at the 6-week follow-up. Five patients developed gout, leading to the discontinuation of BA.

Our data align with previous real-world studies [10,11], indicating that the degree of LDL-c lowering with BA may exceed those reported in phase III randomized control trials (RCTs) [4,5,6]. A pooled analysis of four RCTs evaluating BA in patients with atherosclerotic cardiovascular disease and/or heterozygous familial hypercholesterolemia showed a LDL-c reduction of 17.8% for patients on statins and 24.5% for statin-intolerant patients after 12 weeks of treatment [16]. Several factors may explain these discrepancies: first, the heterogeneity in LDL-c lowering with BA may be influenced by genetic variations in the ACLY gene [17], as well as polymorphisms in other genes involved in cholesterol homeostasis [18]; second, the inter-patient variability in BA pharmacokinetics [19]; finally, differences in patient adherence to diet, lifestyle interventions, and medications, all of which contribute to variability in LDL-c levels [20]. In terms of safety, we observed a lower percentage of adverse events leading to treatment discontinuation compared to previous real-world studies. Our results also differ from those reported in RCTs [21]. Myalgia was the most common side effect, followed by gastrointestinal complaints, which in our study were mainly due to the combination of ezetimibe and BA [22]. Increased uric acid serum levels did not lead to gout, and the levels normalized upon BA discontinuation. The once-daily administration and favorable safety profile of BA contributed to high patient adherence and persistence to treatment, higher than those reported for other lipid-lowering therapies [23,24]. However, we recommend appropriate laboratory and clinical assessment in order to early detect potential side effects and to optimize treatment persistence. In our clinical practice, BA was mainly prescribed to patients at high and very high cardiovascular risk who did not achieve the LDL-c therapeutic target despite maximally tolerated statins dosage and ezetimibe. Among them, BA led to a 28% reduction in serum LDL-c levels and led 53% of patients to achieve the therapeutic LDL-c c target for their risk level.

Recently, Lincoff et al. showed that cardiovascular risk reduction with BA was comparable to that of statins for a given absolute reduction of LDL-c lowering [25]. Because we observed a mean reduction in LDL-c of 33.9 mg/dL (0.876 mmol/L), by using the 2010 Cholesterol Treatment Trialists’ Collaboration meta-analysis [26], we can estimate a relative reduction risk in major cardiovascular events of 19% yearly.

This approach, based on international recommendations [27,28], allowed us to avoid prescribing high-cost lipid-lowering therapies for half of the study population, potentially reducing healthcare spending in the Campania region. Our findings demonstrate a potential cost savings of 33.4% by incorporating BA as a preliminary step in the treatment regimen before resorting to more expensive PCSK9i.

### Limitations

This study has some limitations. First, the small cohort of patients from a single region in Italy, which may limit the generalizability of our findings. Second, the short follow-up period, which may restrict our ability to evaluate long-term effectiveness and safety outcomes. Third, the lack of control group, consequently we could not assess the placebo effect or perform statistical adjustments for potential confounders. Despite these limitations, our study provides valuable insights into the clinical utilization, performance, adherence, and cost-effectiveness of BA in routine practice. Larger European studies with longer follow-up observation time are currently lacking.

## 5. Conclusions

In a real-world setting, BA was mainly prescribed to patients with dyslipidemia at heightened CV risk who had not achieved LDL-c therapeutic targe despite using the maximum tolerated statins dosage and ezetimibe, and to one-third of the patients enrolled due to statin intolerance. BA led to a 28% reduction in LDL-c in patients on high-intensity statins, and a 45% reduction in statin-intolerant patients. Approximately 54% of patients treated with BA reached their LDL-c therapeutic target. Our findings demonstrate a potential cost savings of 33.4% incorporating BA as a preliminary step in the treatment regimen before more expensive drugs. BA was safe, well-tolerated, and characterized by high adherence and persistence.

## Figures and Tables

**Table 1:** Baseline clinical features of the study cohort.

Characteristics	*n* = 111
Age (yrs), mean ± SD	65 ± 9
Gender (male), *n* (%)	68 (61.2)
BMI (kg/m^2^) mean ± SD	27.1 ± 2.3
Smokers, *n* (%)	34 (30.6)
Hypertension, *n* (%)	92 (82.8)
Chronic coronary syndrome, *n* (%)	74 (66.7)
Recent ACS (<1 years), *n* (%)	10 (9.0)
Heart failure with reduced EF, *n* (%)	2 (1.8)
Peripheral artery disease, *n* (%)	25 (22.5)
Previous stroke/TIA, *n* (%)	2 (1.8)
CKD, *n* (%)	8 (7.2)
COPD, *n* (%)	22 (19.8)
Type 2 diabetes mellitus, *n* (%)	22 (19.8)
Type 1 diabetes mellitus, *n* (%)	11 (9.9)
Charles Comorbidity Index, median [IQR]	4.0 [3.0–5.0]
EF (%), mean ± SD	55 ± 7
eGFR (mL/min), mean ± SD	88 ± 28
**Drug Treatment**	
ACE-i/ARB, *n* (%)	96 (86.4%)
Beta-blockers, *n* (%)	33 (75%)
Diuretics, *n* (%)	34 (29.7%)
Statins, *n* (%)	78 (70.3%)
Ezetimibe, *n* (%)	96 (86.5%)
PCSK9i, *n* (%)	15 (13.5%)

SD: standard deviation; BMI: body mass index; TIA: transient ischemic attack; CKD: chronic kidney disease; COPD: chronic obstructive pulmonary disease; ACS: acute coronary syndrome; EF: ejection fraction; IQR: interquartile range; eGFR: estimated glomerular filtration rate; ACE-i/ARB: angiotensin-converting enzyme inhibitors/angiotensin receptor blockers;; PCSK9i: proprotein convertase subtilisin/kexin type 9 inhibitor.

## Data Availability

The data that support the findings of this study are available on reasonable request from the corresponding author.
